# Population health diagnosis with an ecohealth approach

**DOI:** 10.1590/S0034-8910.2015049005842

**Published:** 2015-10-19

**Authors:** Luz Arenas-Monreal, Marlene Cortez-Lugo, Irene Parada-Toro, Lilian E Pacheco-Magaña, Laura Magaña-Valladares

**Affiliations:** I Centro de Investigaciones en Sistemas de Salud. Instituto Nacional de Salud Pública. México; II Centro de Investigaciones en Salud Poblacional. Instituto Nacional de Salud Pública. México; III Secretaria Académica. Instituto Nacional de Salud Pública. México

**Keywords:** Diagnosis of Health Situation, Rural Population, Consumer Participation, Equity, Interdisciplinary Communication, Interpersonal Relations, Gender and Health, Holistic Health, Ecohealth Approach

## Abstract

**OBJECTIVE:**

To analyze the characteristics of health diagnosis according to the ecohealth approach in rural and urban communities in Mexico.

**METHODS:**

Health diagnosis were conducted in La Nopalera, from December 2007 to October 2008, and in Atlihuayan, from December 2010 to October 2011. The research was based on three principles of the ecohealth approach: transdisciplinarity, community participation, gender and equity. To collect information, a joint methodology and several techniques were used to stimulate the participation of inhabitants. The diagnostic exercise was carried out in five phases that went from collecting information to prioritization of problems.

**RESULTS:**

The constitution of the transdisciplinary team, as well as the participation of the population and the principle of gender/equity were differentials between the communities. In the rural community, the active participation of inhabitants and authorities was achieved and the principles of transdisciplinarity and gender/equity were incorporated.

**CONCLUSIONS:**

With all the difficulties that entails the boost in participation, the incorporation of gender/equity and transdisciplinarity in health diagnosis allowed a holistic public health approach closer to the needs of the population.

## INTRODUCTION

The diagnosis of population health is the fundamental tool of research for public health. It allows the identification of the population’s needs and resources available to propose viable solutions to their problems. This diagnosis traditionally included three principles: (1) needs of the population, social and health-disease problems (risks, morbidity and mortality); (2) social determinants; and (3) resources and services.^24^

Two perspectives exist to perform health diagnosis for the population: one is linked to health services; and the other integrates the participation of the population.

Testa^26^ mentions three types of health diagnosis of the population from the point of view of health services: administrative, focused on the analysis of the actions carried out by the institutions on the issue of health; strategic, focused on change, depending on the interests and conflicts that appear on social forces; and ideological, focused on the legitimation resulting from the link between the social forces structured around health with society as a whole. The Pan American Health Organization indicated in recent decades the need to perform health diagnosis with the participation of the population.^25^

Lang and Rayner^16^ analyze public health models throughout history: environmental-sanitarian: biomedical; social; techno-economic; and they also propose a fifth model, the ecological. The health of populations depends on the coexistence between humanity and the physical and social medium in this latest model. To this end, public health must integrate other approaches of thought, such as complex systems, holistic vision and interdisciplinary, in which several agents who consider the multiple dimensions of health converge.^16^ This model coincides with the ecohealth approach.^6,17^ Lebel^17^ defines the ecosystemic or ecohealth approach indicating that strong links between human beings exist, the biophysical environment and socioeconomic aspects reflected in the health of people.

The ecohealth approach was used for problems linked with health and environment,^14,20^ and also with vector-borne diseases.^11,21^ However, no reports around the population health diagnoses based on this approach exist. It promotes the active participation of the population to identify and understand the problems, to establish all the perspectives of the population, the authorities and the research team. Participation boost has a previous background in the Latin American context within proposals of participatory action research and popular education.^12,13^

In this study, we considered the three principles presented by Lebel for the health diagnosis with the ecohealth approach: transdisciplinarity, gender and equity, and community participation.^17^

The aim of this study was to analyze the characteristics of health diagnosis according to the ecohealth approach in rural and urban communities in Mexico.

## METHODS

Health diagnosis were carried out in the following locations in Mexico: La Nopalera, from December 2007 to October 2008, and Atlihuayan, from December 2010 to October 2011.

The team that conducted the health diagnoses had educational purposes and was formed by teachers and students of both sexes, of the master’s program in public health from the Instituto Nacional de Salud Pública (INSP). The academic profile of the team members included the following areas: medicine, nursing, epidemiology, environmental engineering, anthropology, human nutrition, psychology, biochemistry, and education.

Local authorities also participated (ejido commissioner and municipal assistants), teachers of schools, religious representatives and popular groups (organization of farmers, and women in the program “Oportunidades”,^a^ and also students and their families).

Both localities are in the municipality of Yautepec, in a region of central Mexico. The municipality had, in 2010, a population of 97,827 inhabitants. At the time of the health diagnosis, 595 inhabitants were from La Nopalera, and 2,992 were from the neighborhood of Atlihuayan.

The Yautepec River is the main surface current and water source, and it is born in springs near the municipal headwaters. The river is used for agricultural purposes.

La Nopalera was founded at the beginning of 1900. The base of its economy is seasonal agriculture – sorghum and corn –, the form of land ownership is ejidal (street division system), and the house do not have running water. A mobile unit of State health services visit the community once a week.

Atlihuayan is an urban neighborhood of the outskirts of the city. In the past, in this place lands were planted with sugar cane, but in the early 1980s, the sale of land began, and the place turned into a settlement of houses, where families from other regions of the country established themselves. The main occupation was made by employees of offices and shops, construction workers and agricultural workers. The inhabitants receive medical attention at the health center, located ten minutes away by public transportation.

The health diagnosis had five phases:

1. Collecting information in secondary sources: Analysis of demographic, economic, historical, environmental and health damage information data in the municipality. We consulted databases of secondary sources of local history books and records of State and national health services.
2. Approach: The team met with municipal and health authorities to reach an agreement on the health diagnosis of the population. A reconnaissance of the area was also performed. From these activities, it has become easier to contact with residents and local authorities, as well as to identify physical, environmental and social conditions in the region.

Informational meetings were held about the health diagnosis with the various groups of the population (men and women, adolescents, boys and girls). The team performed intentionally meetings with men and women in these sessions. The group was also present in the assemblies with countrymen; went to schools to talk to children and adolescents; and participated in meetings in the church and on health, with women. The educational material used intended to encourage collective reflection about health diagnosis. These activities stimulate the interest and participation of groups of the population, especially the rural community.

3. Diagnosis (compilation of information): A mixed methodology was used (quantitative and qualitative).

The quantitative component included:

1. Formats to collect information from secondary sources: causes of mortality in the civil registry of the municipality (review of death certificates from 1986 to 2010 to analyze the trend of causes of mortality); demographic data (taken from publications of the National Institute of Statistics and Geography and of the National Population Council); causes of demand for consultation of national and state Health Services.
2. A questionnaire with the sections: demographic data; characteristics of housing and public services; morbidity and mortality; access to and use of health services; needs, social and health problems. The questionnaire was applied in all households of the rural community and in a representative sample of the urban community, obtained by the calculation of sample size for global data for finite populations, resulting in 187 families (94% confidence level).

The team applied the questionnaire to the head of the household by home visits in both localities.

The qualitative component included:

1. An ethnographic record of facts and events presented during the team’s participation in different assemblies or meetings.
2. Social cartography: tool for the collective construction of knowledge and part of the premise that it is the locals who know better their own territory.^1^ The questionnaire was carried out with children, adolescents and adults of both genders. Each group drew a map of the location and identified the places that hinder or promote the population’s health. In the end, a reflection was performed with each group about actions that could be undertaken to strengthen the population’s health.
3. Community assembly “festivity”. The entire population (children, adolescents and adults) was invited to identify needs and problems of the community. However, because residents appreciate being invited to a party, the format after the assembly was of “socializing” with custom invitations for each family. To inquire about their needs and problems, several techniques were used, taking age into account: with adult men and women, by group reflections; with adolescents, a board game was held and some participated in the graphic record (photos and videos); with children we used children’s games. The activities counted with the participation of local authorities.

The team enabled the reflection on the bond of the social and health problems with the physical, social and economic environment to deepen their understanding.

All the assistants and the team consumed food and beverages from the region during their stay.

The residents of urban areas and local authorities did not attend the community meetings.

4. Prioritization phase:

1. Problem identification. A second community meeting (“socializing”) was convened in the rural community by means of invitations addressed to the people who participated in the previous meeting. Seven working groups were formed. Each selected two representatives to identify the top ten problems of the community. We used the method of prioritizing by Hanlon (adapted), with the criteria of magnitude, transcendence, feasibility and vulnerability (Table 1).
2. Problem analysis and prioritization: Representatives of various groups, designated in the identification of problems in the rural community, were summoned. The participants analyzed the prioritized problems based on five components: social and environmental determinants; health effects; responsible for the solution of these problems (individual, family, community, society); responsibility of the population; solution viability. Reflections on the interaction between health problems and the social and environmental aspects of the region were encouraged. Authorities, population and research team elected five problems that can be addressed based on these elements.

**Table 1:** Prioritization with the method adapted from Hanlon. Nopalera and Atlihuayan (Mexico), 2007 to 2011.

Criterion	Score			
Magnitude: Who does it affect? (people)	Very few	Few	Most	
Score	1	3	5	
Transcendence: What is the gravity? (severe)	It is not	Little	Serious	Very serious
Score	0	2	4	5
Feasibility: To what extent is it possible to solve the problem?				
a. Relevant: This is the right time to do something about this problem?	Yes	No		
Score	1	0		
b. Economical: Are there economic resources (money) to solve this problem?	Yes	No		
Score	1	0		
c. Acceptability: The community would feel comfortable working with this theme?	Yes	No		
Score	1	0		
d. Resources: Are there people and materials in the community to work with this problem?	Yes	No		
Score	1	0		
e. Legality: Is there any law or agreement in the community that would prevent work with this theme?	Yes	No		
Score	1	0		
Vulnerability: What is the level of difficulty of this problem?	Very difficult	Difficult	Easy	
Score	1	3	5	

The prioritization exercise in the urban community was carried out with the local authority and six members of the community.

4. Return of results: The results were presented in a meeting with the population, and local and municipal authorities of the rural community. From these results, collective decisions were taken to develop community initiatives to address some of the problems identified in the health diagnosis.

The assistance of the population was scarce in the urban area and the diagnosis was delivered to local and municipal authorities.

We performed descriptive analysis of sociodemographic characteristics of each community and of the characteristics of interest for methodological evaluation of the health diagnosis with an ecosystemic approach. Descriptive analyses were conducted using the statistical package Stata (v 12.1; Stata Corp., College Station, TX, USA).

We transcribed ethnographic registry notes, workshops, assemblies and social mapping in Word^®^ processor v. 2007, and subsequently converted them to the Ethnograph v.5 calculation program.

The information was triangulated with quantitative and qualitative data.

The health diagnosis protocol was approved by the colleges of education and Social Sciences of INSP on its eighth ordinary session of September, 2007. We requested the consent of all inhabitants of the community to carry out each activity.

## RESULTS

The rural community participated increasingly in each phase of the diagnosis in the same way that local authorities. However, difficulties to participate were detected in the urban community at all stages. It was difficult to establish contact with the population in the stage of approximation, and it was only possible to work with some members of community organizations. Urban authorities of the urban community showed little interest in the diagnosis (Table 2).

**Table 2:** Results of the health diagnosis in the rural community La Nopalera and urban community Atlihuayan (Mexico), 2007 to 2011.

Community	Phase	Population group
La Nopalera	Approach	30 ejido farmers, 10 women of the Church, 120 adult women, 180 students, municipal assistant, and ejidal commissioner
	Diagnosis	All the families were visited and the questionnaire was applied, an exercise in cartography was performed with 8 adults (men and women), high school students and children from the 5^th^ and 6^th^ grades of elementary school. 90 people attended the diagnostic assembly (children), young people and adults, including local authorities
	Prioritization/Problematization	A community assembly of prioritization with average assistance of 130 people (children, adolescents and adults). A problematization exercise with 10 community representatives elected at the previous meeting. Including local authorities
	Return of results	Community assembly with 60 people. The delivery was made to local authorities/Results were delivered to municipal authorities
Atlihuayan	Approach	80 adult women, 30 people of the Church
	Diagnosis	187 families were visited for the application of the questionnaires (187 of 731 families), cartography exercise with 6 adults and 20 children
	Prioritization/Problematization	Did not attend community assemblies and the prioritization exercise was carried out with local authorities and members of the community; 6 people were present
	Return of results	20 adults attended the meeting for return of results, and the diagnosis document was delivered to local authorities

The sociodemographic characteristics were similar in the distribution by sex in both localities, with an average age of 28 years and a greater percentage of people with higher education levels in the urban community. We noticed a higher percentage of people dedicated to agricultural activities in the rural community (Table 3). There were differences in the level of marginalization and in running water allocation between the communities (Table 4).

**Table 3:** Demographics characteristics of participants of each community. La Nopalera and Atlihuayan (Mexico), 2007 to 2011.

Characteristic	Community			
	La Nopalera		Atlihuayan	

	n	%	n	%
Gender				
Female	309	51.9	400	51.0
Male	286	48.1	384	49.0
Age (years)				
Average	28.3		27.8	
Rango	0 - 100		0 - 91	
Age by groups				
0 to 5	65	10.9	105	13.4
6 to 17	188	31.6	200	25.5
18 to 25	66	11.1	107	13.6
26 to 40	114	19.2	170	21.7
41 to 60	98	16.5	130	16.6
> 60	64	10.7	72	9.2
Educational Level^a^				
None^b^	45	8.6	5.4	8.0
Elementary School	259	49.6	250	37.1
High School	163	31.2	213	31.6
Preparatory	37	7.1	127	18.8
Teacher’s training degree	18	3.5	30	4.5
Occupation				
Home	145	26.8	170	24.3
Student	178	32.9	212	30.3
Farmer	105	19.4	31	4.5
Construction worker	11	2.1	47	6.7
Employee	43	7.9	103	14.7
Businessman^c^	26	4.8	48	6.9
Unemployed	13	2.4	17	2.4
Other	20	3.7	71	10.2

a Complete and incomplete education levels.

b ≥ 18 years.

c Formal and informal.

**Table 4:** Profile of the studied communities according to the health diagnosis. La Nopalera and Atlihuayan (Mexico), 2007 to 2011.

Community	La Nopalera	Atlihuayan
Number of households interviewed	143	200
Marginalization	High	Low
Illiteracy (%)^a^	12.1	9.5
Running water (%)	0	91.0
Sewage treatment (%)	86.0	89.5
Garbage collection by truck	74.8	80.0
Dirt floor (%)	31.5	28.0
Electricity (%)	97.9	98.0
Agglomeration (%)^b^	52.4	40.0
International migration of at least one member of each family (%)	34.3	16.5

a ≥ 15 years.

b 2.5 people per room.

The results of the prioritization, according to the method adapted from Hanlon, to the rural community were: disease (intestinal parasitosis, dengue fever, diabetes mellitus) and social and environmental determinants (garbage, river contamination, water shortages, unemployment and lack of opportunities for young people). To the urban community, they were: social and environmental determinants (garbage, river contamination, social insecurity, alcoholism, violence) (Data not shown).

The transdisciplinary approach held in both communities had different results. It was possible to integrate the vision of the population and the sociopolitical agents in the rural community; while it was not possible to incorporate local authorities or even the entirety of the inhabitants in the urban community.

Men and women of the rural community attended all activities. Health problems seen as integral to the interaction with the physical and social environment has aroused similar interest in children, adolescents and adults of both genders. This fact made it possible to obtain the perspective of men and women in the various activities, in the social mapping and in the prioritization exercises.

The participation of men in urban community was minimal, so the groups were mostly of adult women. A mixed group was achieved only in the cartography exercises with boys and girls. In this community, no groupings of men existed (e.g., grouped by profession, sport, culture, among others), one of the reasons that hindered their integration in the different stages of the diagnosis.

The urban population lacked community belonging and had better conditions of basic consumption, compared to the countryside. These circumstances were translated into disinterest and lack of commitment of the inhabitants to improve their environment.

The ecohealth approach has helped the population to visualize the relationship between the health, the physical environment and socioeconomic aspects, and to identify solutions. The participation of local authorities (at least in the rural community) favored strategies for finding solutions.

In the countryside, the assemblies contributed to generate educational processes with the population, to advance the understanding of the health-disease phenomenon, that will not be visualized, but in the interaction with the sociocultural, economic and environmental aspects.

The generation of knowledge from the principles of the ecohealth approach allowed the development of proposals according to the themes prioritized by the population. Community initiatives in both localities focused on: solid waste management,^b,c^ control of “flies” of porcine farm,^d^ children and young people with gender perspective,^e^ strengthening of community belonging for children and their families^f^ and addiction prevention for children and adolescents.^g^

## DISCUSSION

Health diagnosis results, with respect to the participation of the population were different in both localities (with low participation in the urban community). However, in studies based on the ecohealth approach, in which specific problems were addressed, we obtained good results by boosting the participation of the population.^4,11,20^ This difference in participation in the diagnoses may be related to low community sense of belonging of the population in the urban area. Chavis et al^7,8^ gave solid support, showing the relationship between the sense of community and belonging, and community participation. Other authors confirm what Chavis et al^18^ reported, pointing out that, in so far as the community sense of belonging contributes to participation, these elements together strengthen the construction of citizenship.^23^

The participation of the population was favorable in the rural area, similar to what was reported in a study with older adults in Chile.^15^

In this investigation, we observed that local authority in the urban community participated and committed little, which is also found in another study of the ecosystemic approach conducted on Ecuador.^3^ We agree with these authors, who point out that political culture and context are factors that influence the way authorities act.

The participation of the population in the health diagnosis lies at the local level and its importance is the identification of the involved of their own problems and respective solutions. Menéndez mentions that the sociopolitical and economic decisions that affect the lives of people are taken by political bodies, authorities, and institutions without the participation of the major social groups.^19^ However, participation at the local level is a process that can contribute to the construction of citizenship and to strengthen social agents so that they negotiate with the State,^5^ not denying the difficulties and conflicts of power that may arise during the process between the population and the State.

The population was able to identify, prioritize and engage with the solution of health problems in the countryside. The health diagnosis with the ecohealth approach establishes a substantial difference regarding diagnoses of traditional health (administrative, strategic and ideological), from health services, and are focused on goals, objectives and programming activities determined by experts, addressed to the people, but without its participation.^24,26^

The results rely on transdisciplinarity, participation, and equity/gender. The also agree with Dakubo’s^9^ observations, which state that the current approach of public health is benefited with the implementation of a holistic perspective that incorporates the complexity of health-disease process.

Dakubo^10^ points out that research based on communities is a model that drives the participation of the population, authorities and research team in the various phases of the project. For health diagnosis in this study, we considered elements of the research in the community, but it was not possible for the urban population to participate at all stages. A tool used to promote the participation of the population was social cartography. Its use has allowed promoting participation and improving the understanding of the local context by inhabitants, thus finding solutions to their problems.^2^

The ecohealth approach discusses equity and gender as a single principle. First, because it is not possible to speak of equity without addressing gender, and vice versa. Second, because to leave these themes aside is to partially understand the health status of the communities.^6,17^ The vision of men and women has been integrated to rural community health diagnosis, and partly in the urban community.

Mertens et al^20^ believe that the gender is a category that shows in a differential manner the way by which men and women interact with the environment and with health, and the manner in which they participate in the projects. This coincides with the results of this research.

The challenges identified were: a) to integrate in the prioritization tool problems of environmental determinants to reflect together with the population on relations and social-ecological interactions that affect health, well-being and environmental sustainability; b) to draw strategies and mechanisms which enable the boosting of the participation of the population in several geographical areas (urban/rural) and in different social and cultural contexts; c) to raise awareness of those who make decisions of health services about the importance of conducting population health diagnosis with this approach; d) to promote the integration not only of local and municipal authorities, but also of national and state institutions for health diagnosis with the ecohealth approach, to influence public policy development.

The health diagnosis of the population is the primary tool for health personnel and health authorities to make decisions, often limited by the obtaining of data (statistics from health services, without regard to the population). We went from the premise of health diagnosis, with an ecohealth approach, as a first step to seek an approximation with the populations and to generate knowledge that will lead to actions that are integrated and that responds to their needs.

This study is a first approximation of a health diagnosis based on the ecohealth approach. It is necessary to continue conducting such diagnosis, setting the strategies according to the characteristics of the population and of the geographical areas, to deepen the concretion of the three principles of the approach presented in this paper.

## Footnotes

a “Oportunidades” is a Mexican program focused on poor families that supports education, health and nutrition. The receiver of the resources is the mother of the family.

b Domínguez-Ortiz MG. Separación de los residuos sólidos urbanos, susceptibles a ser valorizados, que contribuya al mejoramiento del entorno de la colonia de Atlihuayan, municipio de Yautepec Morelos, bajo el enfoque ecosistémico [thesis]. Cuernavaca: Escuela de Salud Pública de México/Instituto Nacional de Salud Pública; 2012.

c Pacheco-Magaña LE. Manejo integral de los residuos sólidos para contribuir al control de enfermedades asociadas a estos, en la población de la localidad La Nopalera, Yautepec, Morelos bajo el enfoque de monitoreo participativo [thesis]. Cuernavaca: Escuela de Salud Pública de México/Instituto Nacional de Salud Pública; 2009.

d González C. Implementación de control biológico de moscas y propuesta para el manejo de excretas en la granja porcina de La Nopalera, Yautepec, Morelos [thesis]. Cuernavaca: Escuela de Salud Pública de México/Instituto Nacional de Salud Pública; 2010.

e Espinosa-Cárdenas F. Sexualidad y perspectiva de género: intervención educativa con jóvenes de La Nopalera [thesis]. Cuernavaca: Escuela de Salud Pública de México/Instituto Nacional de Salud Pública; 2009.

f Camarillo-Elizalde DG. Promoción de la salud y pertenencia comunitaria en escolares de Atlihuayan, Morelos [thesis]. Cuernavaca: Escuela de Salud Pública de México/Instituto Nacional de Salud Pública; 2012.

g Nieto Ayala M. Habilidades para la vida: prevención de adicciones en adolescentes de la telesecundaria Belisario Dominguez de Atlihuayan, Yautepec, Morelos [thesis]. Cuernavaca: Escuela de Salud Pública de México/Instituto Nacional de Salud Pública; 2012.
