# Supplementary material for: Population health diagnosis with an ecohealth approach
Source: Rev Saude Publica. 2015 Oct 19;49:78. doi: 10.1590/S0034-8910.2015049005842 (PMC4617434; doi:10.1590/S0034-8910.2015049005842)
Supplement: Translation

# Translation: Diagnóstico de salud poblacional con enfoque de ecosalud

**Authors:** Luz Arenas-Monreal, Marlene Cortez-Lugo, Irene Parada-Toro, Lilian E Pacheco-Magaña, Laura Magaña-Valladares

**Affiliations:** I Centro de Investigaciones en Sistemas de Salud. Instituto Nacional de Salud Pública. México; II Centro de Investigaciones en Salud Poblacional. Instituto Nacional de Salud Pública. México; III Secretaria Académica. Instituto Nacional de Salud Pública. México

**Keywords:** Diagnóstico de la Situación de Salud, Población Rural, Participación Comunitaria, Equidad, Comunicación Interdisciplinaria, Relaciones Interpersonales, Género y Salud, Salud Holística, Enfoque de Ecosalud

## Abstract

**OBJETIVO:**

Analizar las características del diagnóstico de salud según el enfoque de ecosalud en comunidades rural y urbana en México.

**MÉTODOS:**

Los diagnósticos de salud se efectuaron en La Nopalera, de diciembre 2007 a octubre 2008 y en Atlihuayan de diciembre 2010 a octubre de 2011. Se basó en tres principios del enfoque de ecosalud: transdiciplina, participación comunitaria, género y equidad. Para colectar la información se utilizó una metodología mixta y diversas técnicas para estimular la participación de los habitantes. El ejercicio de diagnóstico se efectuó en cinco fases que oscilaron desde la recolección de información hasta la priorización de problemas.

**RESULTADOS:**

La constitución del equipo transdisciplinario, así como la participación de la población y el principio de género/equidad fue diferencial entre las comunidades. En la comunidad rural, se logró la participación activa de los pobladores y autoridades y se incorporaron los principios de transdisciplina y género/equidad.

**CONCLUSIONES:**

Con todas las dificultades que acarrea el impulso de la participación, la incorporación del género/equidad y la transdisciplina en el diagnóstico de salud, permitió un abordaje holístico de salud pública más cercano a las necesidades de la población.

## INTRODUCCIÓN

El diagnóstico de salud poblacional es la herramienta fundamental de investigación en salud pública. Permite identificar las necesidades de la población y los recursos con que cuenta para proponer soluciones viables a sus problemáticas. Este diagnóstico, tradicionalmente, ha incluido tres ejes: (1) necesidades de la población, problemas sociales y de salud-enfermedad (riesgos, morbilidad y mortalidad), (2) determinantes sociales y (3) recursos y servicios.^24^ La salud pública, además de enfocarse en riesgos y daños, debe incluir aspectos positivos, con lo cual surge otro eje que se puede incluir en el diagnóstico: (4) las fortalezas de la población.^22^

Existen dos perspectivas para efectuar diagnóstico de salud poblacional: una se vincula con los servicios de salud; otra integra la participación poblacional.

Testa^26^ menciona tres tipos de diagnóstico de salud poblacional desde la óptica de los servicios de salud: administrativo, dirigido al análisis de las acciones que efectúan las instituciones frente a la problemática de salud; estratégico, enfocado en el cambio, en función de los intereses y conflictos que aparecen en las fuerzas sociales; e ideológico, dirigido a la legitimación resultante del vínculo entre las fuerzas sociales estructuradas en torno a la salud con el conjunto de la sociedad. La Organización Panamericana de la Salud señaló en décadas recientes la necesidad de efectuar diagnósticos de salud con la participación de la población.^25^

Lang y Rayner^16^ analizan los modelos de salud pública a lo largo de la historia: sanitarista-ambiental; biomédico; social; tecno-económico; y proponen un quinto modelo, el ecológico. La salud de las poblaciones depende de la coexistencia entre la humanidad y el ambiente físico y social en este último modelo. Para ello, la salud pública debe integrar otros abordajes de pensamiento, como sistemas complejos y visión holística e interdisciplinariedad en las que confluyan diversos actores que consideren las múltiples dimensiones de la salud.^16^ Este modelo posee coincidencias con el enfoque de ecosalud.^6,17^ Lebel^17^ define el enfoque ecosistémico o de ecosalud al indicar que existen fuertes vínculos entre los seres humanos, el ambiente biofísico y los aspectos socioeconómicos que se reflejan en la salud de las personas.

El enfoque de ecosalud ha sido utilizado en problemáticas vinculadas con la salud y el ambiente,^14,20^ y con enfermedades transmitidas por vector.^11,21^ Sin embargo, no existen reportes en torno a diagnósticos de salud poblacional con base en este enfoque. Este enfoque promueve la participación activa de la población en la identificación y comprensión de los problemas, al conjuntar las perspectivas de población, autoridades y equipo investigador. El impulso de la participación tiene antecedentes previos en el contexto latinoamericano dentro de las propuestas de investigación-acción-participativa y en educación popular.^12,13^

Se consideraron los tres principios planteados por Lebel para el diagnóstico de salud con enfoque de ecosalud en este estudio: transdisciplina, género y equidad y participación comunitaria.^17^

El objetivo de este estudio fue analizar las características del diagnóstico de salud según el enfoque de ecosalud en comunidades rural y urbana en México.

## MÉTODOS

Los diagnósticos de salud se efectuaron en las localidades de México: La Nopalera, de diciembre 2007 a octubre 2008 y en Atlihuayan, de diciembre 2010 a octubre de 2011.

El equipo que realizó los diagnósticos de salud tuvo finalidad pedagógica y se constituyó por profesoras y estudiantes de ambos sexos, pertenecientes a la maestría en salud pública del Instituto Nacional de Salud Pública (INSP). El perfil de los integrantes del equipo estaba enmarcado en las áreas de: medicina, enfermería, epidemiología, ingeniería ambiental, antropología, nutrición humana, psicología, bioquímica y educación.

Participaron también autoridades locales (comisariado ejidal, ayudantes municipales), docentes de escuelas, representantes religiosos y grupos poblacionales (organización campesina y mujeres del programa “Oportunidades”,^a^ agrupaciones religiosas de madres y padres de familia de las escuelas y estudiantes).

Ambas localidades se ubican en el municipio de Yautepec, en una región del centro de México. El municipio tenía, en 2010, una población de 97.827 habitantes.^b^ Al momento de los diagnósticos de salud, 595 habitantes eran de La Nopalera, y 2.992, del barrio de Atlihuayan.

El río Yautepec es la principal corriente superficial de agua y nace en manantiales cercanos a la cabecera municipal. El río se utiliza para fines agropecuarios.

La Nopalera se fundó a principios de 1900. La base de su economía es la agricultura de temporal – sorgo y maíz –, la tenencia de la tierra es ejidal, las viviendas no cuentan con agua entubada. Una unidad móvil de servicios de salud estatal acude a la comunidad una vez por semana.

Atlihuayan es un barrio urbano periférico del municipio. Antiguamente en ese lugar existían tierras ejidales sembradas de caña de azúcar, pero en la década de los 80’s se inició la venta de tierras y se transformó en un asentamiento de viviendas, en el que se establecieron familias de otras regiones del país. La ocupación principal es por empleados de oficinas y comercios, obreros de la construcción y trabajadores agrícolas. Los habitantes acuden al centro de salud para recibir atención médica, ubicado a 10 minutos en transporte público.

El diagnóstico de salud tuvo cinco fases:

1. Recolección de información en fuentes secundarias: Análisis de información de datos sociodemográficos, económicos, históricos, ambientales y de daños a la salud en el municipio. Se consultaron bases de datos de fuentes secundarias, libros de historia local y registros de los servicios de salud estatales y nacionales.
2. Acercamiento: El equipo se entrevistó con autoridades municipales y sanitarias a fin de acordar el objetivo del diagnóstico de salud poblacional. Realizó, además, recorridos en la zona. A partir de estas actividades, se facilitó el contacto con habitantes y autoridades locales, así como la identificación de las condiciones físico-ambientales y sociales de la región.

Se sostuvieron reuniones informativas acerca del diagnóstico de salud con los diferentes grupos poblacionales (hombres y mujeres adultas, adolescentes, niños y niñas). El equipo realizó intencionalmente reuniones con hombres y mujeres en estas sesiones. Se acudió a las asambleas con los campesinos; se fue a las escuelas, con los niños y adolescentes; y a reuniones en la iglesia y de salud, con las mujeres. Se utilizó material educativo destinado a alentar la reflexión colectiva acerca del diagnóstico de salud. Estas actividades estimularon el interés y la participación de los grupos poblacionales, en especial en la comunidad rural.

3. Diagnóstico (recolección de información): Se utilizó metodología mixta (cuantitativa y cualitativa).

El componente cuantitativo incluyó:

- Formatos para recabar información de fuentes secundarias: causas de mortalidad en el registro civil del municipio (revisión de certificados de defunción de 1986 a 2010 para analizar la tendencia de las causas de mortalidad); datos sociodemográficos (recabados de publicaciones del Instituto Nacional de Estadística y Geografía y del Consejo Nacional de Población); causas de demanda de consulta, morbilidad, recursos (físicos, humanos, económicos e instituciones) recabados de los Servicios de Salud estatales y nacionales.
- Cuestionario con las secciones: datos sociodemográficos; características de la vivienda y los servicios públicos; morbilidad y mortalidad; acceso y uso de servicios de salud; necesidades, problemas sociales y de salud y fortalezas. Se aplicó el cuestionario en todas las viviendas de la localidad rural, y en una muestra representativa de la urbana, obtenida mediante el cálculo de tamaño de muestra para datos globales para poblaciones finitas, resultando en 187 familias (nivel de confianza de 94%).

El equipo aplicó el cuestionario al jefe o jefa de familia, mediante visitas domiciliarias en ambas localidades.

El componente cualitativo consistió en:

- Registro etnográfico de los hechos y eventos que se presentaron durante la estancia del equipo y en las diferentes asambleas o reuniones en las que se participó.
- Cartografía social: herramienta para la construcción colectiva del conocimiento y parte de la premisa que son los propios habitantes quienes conocen mejor su territorio.^1^ Se realizó con niños, adolescentes y adultos de ambos géneros. Cada grupo dibujo un mapa de la localidad e identificaron los sitios que dificultan o favorecen la salud de la población. Al final, se reflexionó con cada grupo sobre las acciones que se podrían hacer para fortalecer la salud poblacional.
- Asamblea comunitaria “festividad”. Se convocó a toda la población (niños, adolescentes y adultos) para identificar necesidades y problemas de la comunidad. Sin embargo, debido a que los pobladores valoran más ser invitados a una festividad, el formato que siguió la asamblea fue de “convivio” con invitaciones personalizadas por familia. Para indagar sus necesidades y problemáticas, se trabajó con diversas técnicas, teniendo en cuenta la edad: con hombres y mujeres adultos, en reflexiones grupales; con adolescentes, se realizó un juego de mesa y algunos participaron en el registro gráfico (fotografías y videos); con los niños hubo juegos infantiles. Se tuvo participación de autoridades locales.

El equipo propició la reflexión sobre el vínculo de los problemas de salud y sociales con el ambiente físico, social y económico a fin de profundizar en su comprensión.

Todos los asistentes y el equipo convivieron consumiendo alimentos y bebidas de la región.

A las asambleas comunitarias no acudieron los habitantes de la zona urbana ni las autoridades locales.

4. Fase de priorización:

- Identificación de problemas. Se convocó a una segunda asamblea comunitaria (“convivio”) en la zona rural a través de invitaciones dirigidas a personas que participaron en la reunión anterior. Se conformaron siete grupos de trabajo. Cada uno seleccionó dos representantes para identificar los diez principales problemas de la comunidad. Se utilizó el método de priorización de Hanlon (adaptado), con los criterios de magnitud, trascendencia, factibilidad y vulnerabilidad (Tabla 1).
- Análisis de problemas y priorización*. *Se convocó a los representantes de cada grupo, designados en la asamblea de identificación de problemas en la zona rural. Los participantes analizaron los problemas priorizados con base en cinco componentes: determinantes sociales y ambientales; efectos para la salud; responsables de la solución de esos problemas (individual, familiar, comunitario, social); responsabilidad de la población; factibilidad de solución. Se alentó la reflexión acerca de la interacción entre los problemas de salud y los aspectos sociales y ambientales de la región. Autoridades, población y equipo investigador eligieron cinco problemas factibles de ser abordados con base en estos elementos.

**Tabla 1:** Priorización con el método adaptado de Hanlon. Nopalera y Atlihuayan (México), 2007 a 2011.

Criterio	Puntaje			
Magnitud: ¿A quién afecta? (personas)	Muy pocas	Pocas	La mayoría	
Puntaje	1	3	5	
Trascendencia: ¿Qué tan grave es? (grave)	No es	Poco	Grave	Muy grave
Puntaje	0	2	4	5
Factibilidad: ¿Qué tan posible es resolver el problema?				
a. Pertinentes: Este es el momento oportuno para hacer algo en relación a este problema?	Sí	No		
Puntaje	1	0		
b. Económico: ¿Existen los recursos económicos (dinero) para resolver este problema?	Sí	No		
Puntaje	1	0		
c. Aceptabilidad: ¿La comunidad se sentiría cómoda de trabajar en este tema?	Sí	No		
Puntaje	1	0		
d. Recursos: Hay gente y materiales en la comunidad para trabajar con este problema?	Sí	No		
Puntaje	1	0		
e. Legalidad: Existe alguna ley o acuerdo en la comunidad que impida trabajar con este tema?	Sí	No		
Puntaje	1	0		
Vulnerabilidad: ¿Qué tan difícil la solución de este problema?	Muy difícil	Difícil	Es fácil	
Puntaje	1	3	5	

El ejercicio de priorización en la zona urbana se efectúo con la autoridad local y seis integrantes de la comunidad.

5. Devolución de resultados. Fueron presentados los resultados en una asamblea con la población, autoridades locales y municipales de la zona rural. A partir de éstos, se tomaron decisiones colectivas para efectuar iniciativas comunitarias para enfrentar algunos problemas identificados en el diagnóstico de salud.

La asistencia poblacional fue escasa en la zona urbana y se entregó el documento del diagnóstico a las autoridades locales y municipales.

Se realizó análisis descriptivo de las características sociodemográficas de cada comunidad y de las características de interés para la evaluación metodológica del diagnóstico de salud con enfoque ecosistémico. Los análisis descriptivos se efectuaron utilizando el paquete estadístico Stata (v12.1; Stata Corp., College Station, TX, USA).

Se transcribieron notas de registro etnográfico, sesiones de trabajo, asambleas y cartografía social en el procesador de textos de Word^®^ v. 2007 que, posteriormente, se transformaron al programa de computo Ethnopraph v.5.

Se trianguló la información con los datos cuantitativos y cualitativos.

El protocolo del diagnóstico de salud fue aprobado por los colegios de educación y ciencias sociales del INSP en su octava sesión ordinaria del mes de septiembre del año 2007. A todos los habitantes de la comunidad se les pidió consentimiento informado para efectuar cada actividad.

## RESULTADOS

La comunidad rural participó de manera creciente en cada fase del diagnóstico, al igual que las autoridades locales. En contraste, se tuvo dificultad para la participación en la localidad urbana en todas las fases. Fue difícil establecer contacto con toda la población en la etapa de acercamiento y sólo se pudo trabajar con algunos integrantes de organizaciones comunitarias. Las autoridades locales de la zona urbana mostraron poco interés en el diagnóstico (Tabla 2).

**Tabla 2:** Resultados del diagnóstico de salud en la comunidad rural Nopalera, y urbana Atlihuayan (México), 2007 a 2011.

Comunidad	Fase	Grupo poblacional
La Nopalera	Acercamiento	30 ejidatarios, 10 mujeres de la iglesia, 120 mujeres adultas, 180 escolares, Ayudante municipal y Comisariado ejidal
	Diagnóstico	Se visitó a todas las familias y se aplicó el cuestionario, ejercicio de cartografía con 8 personas adultas (hombres y mujeres), estudiantes de 1º, 2º y 3º grado de la Telesecundaria local y niños(as) de 5º y 6º de la primaria. A la asamblea de diagnóstico acudieron 90 personas (niños(as), jóvenes y adultos(as)), incluyendo autoridades locales
	Priorización/Problematización	Una asamblea comunitaria de priorización con una asistencia promedio de 130 personas (niños, adolescentes y adultos). Un ejercicio de problematización con 10 representantes comunitarios previamente elegido en la asamblea anterior. Incluyendo autoridades locales
	Devolución de resultados	Asamblea comunitaria con 60 personas y se hizo entrega a autoridades locales/Se devolvieron resultados a las autoridades municipales
Atlihuayan	Acercamiento	80 mujeres adultas, 30 personas iglesia
	Diagnóstico	Se visitó a 187 familias para la aplicación del cuestionarios (187 de 731 familias), ejercicio de cartografía con 6 personas adultas y 20 niños(as)
	Priorización/Problematización	No acudieron a las asambleas comunitarias y el ejercicio de priorización se efectúo con autoridades locales e integrantes de la comunidad en total fueron 6 personas
	Devolución de resultados	Acudieron 20 personas adultas para devolución de resultados y se entregó el documento del diagnóstico a las autoridades locales

Las características sociodemográficas fueron similares en la distribución por sexo en ambas localidades, con edad promedio de 28 años y mayor porcentaje de personas con más escolaridad en la comunidad urbana. Existió mayor porcentaje de personas dedicadas a las actividades agrícolas en la comunidad rural (Tabla 3). Hubo diferencias en el nivel de marginación y la dotación de agua entubada entre las comunidades (Tabla 4).

**Tabla 3:** Características sociodemográficas de los participantes de cada comunidad. Nopalera y Atlihuayan (México), 2007 a 2011.

Característica	Comunidad			
	La Nopalera		Atlihuayan	

	n	%	n	%
Sexo				
Femenino	309	51.9	400	51.0
Masculino	286	48.1	384	49.0
Edad (años)				
Promedio	28.3		27.8	
Rango	0 - 100		0 - 91	
Edad por grupos				
0 a 5	65	10.9	105	13.4
6 a 17	188	31.6	200	25.5
18 a 25	66	11.1	107	13.6
26 a 40	114	19.2	170	21.7
41 a 60	98	16.5	130	16.6
> 60	64	10.7	72	9.2
Nivel educacional^a^				
Ninguna^b^	45	8.6	5.4	8.0
Primaria	259	49.6	250	37.1
Secundaria	163	31.2	213	31.6
Preparatoria	37	7.1	127	18.8
Licenciatura	18	3.5	30	4.5
Ocupación				
Hogar	145	26.8	170	24.3
Estudiante	178	32.9	212	30.3
Campesino	105	19.4	31	4.5
Albañil	11	2.1	47	6.7
Empleado	43	7.9	103	14.7
Comerciante^c^	26	4.8	48	6.9
Desempleado	13	2.4	17	2.4
Otro	20	3.7	71	10.2

a Niveles de educación completos e incompletos.

b ≥ 18 años.

c Formal e informal.

**Tabla 4:** Perfil de las comunidades de estudio de acuerdo con el diagnóstico de salud. Nopalera y Atlihuayan (México), 2007 a 2011.

Comunidad	La Nopalera	Atlihuayan
Números de viviendas entrevistadas	143	200
Marginación	Alta	Baja
Analfabetismo (%)^a^	12.1	9.5
Agua entubada (%)	0	91.0
Alcantarillado (%)	86.0	89.5
Recolección de basura por camión	74.8	80.0
Piso de tierra (%)	31.5	28.0
Electricidad (%)	97.9	98.0
Hacinamiento (%)^b^	52.4	40.0
Migración internacional de al menos un integrante de cada familia (%)	34.3	16.5

a ≥ 15 años.

b 2.5 por cuarto.

Los resultados de la priorización, según el método adaptado de Hanlon, para la comunidad rural fueron: enfermedades (parasitosis intestinales, dengue, diabetes mellitus) y determinantes sociales y ambientales (basura, contaminación del río, falta de agua, desempleo y falta de oportunidades para jóvenes). Para la comunidad urbana, fueron: determinantes sociales y ambientales (basura, contaminación del río, inseguridad social, alcoholismo, violencia) (Datos no mostrados).

El acercamiento transdisciplinario que se realizó en ambas comunidades tuvo resultados diferenciados. Se logró integrar la visión de la población y actores sociopolíticos en la comunidad rural; no fue posible incorporar ni a las autoridades locales ni a todos los habitantes en la localidad urbana.

Hombres y mujeres asistieron a todas las actividades en la localidad rural. La problemática de la salud vista de forma integral en la interacción con el ambiente físico y social despertó interés similar en los niños, adolescentes y adultos de ambos géneros. Esto posibilitó obtener la perspectiva de hombres y mujeres en las distintas actividades, la cartografía social y los ejercicios de priorización.

Fue mínima la participación de los hombres en la localidad urbana y los grupos fueron mayoritariamente de mujeres adultas. Sólo en el ejercicio de cartografía con los niños y niñas se consiguió un grupo mixto. En esta localidad, no existían agrupaciones de hombres (por ejemplo, agrupados por ocupación, deportes, cultura, entre otros), una de las razones que dificultó su integración a las distintas fases del diagnóstico.

La población urbana carecía de pertenencia comunitaria y poseía mejores condiciones de satisfactores básicos, en comparación con la zona rural. Estas circunstancias se tradujeron en desinterés y falta de compromiso de los habitantes para mejorar su entorno.

El enfoque de ecosalud contribuyó para que la población visualice la relación que existe entre los problemas de salud, el ambiente físico y los aspectos socioeconómicos, y para identificar soluciones. La participación de las autoridades locales (por lo menos en la localidad rural) favoreció estrategias para encontrar soluciones.

En la localidad rural, las asambleas (“convivios”) contribuyeron a generar procesos educativos con la población, para avanzar en la comprensión del fenómeno salud-enfermedad y que no se visualizará sólo desde el punto de vista biológico sino en la interacción con los aspectos socio-culturales, económicos y ambientales.

La generación del conocimiento a partir de los principios del enfoque de ecosalud permitió desarrollar propuestas acordes con los temas priorizados por la población. Las iniciativas comunitarias en ambas localidades se enfocaron en: manejo de residuos sólidos,^c,d^ control de “moscas” de granja porcina,^e^ niños y jóvenes con perspectiva de género,^f^ fortalecimiento de pertenencia comunitaria en niños y sus familias^g^ y prevención de adicciones en niños y adolescentes.^h^

## DISCUSIÓN

Los resultados en los diagnósticos de salud, con relación a la participación poblacional fueron diferentes en ambas localidades (con baja participación en la zona urbana). Sin embargo, en estudios efectuados con base en el enfoque de ecosalud, en los cuales se abordan problemáticas específicas, se reportan buenos resultados impulsando la participación de la población.^4,11,20^ Esta diferencia en la participación en los diagnósticos, puede relacionarse con la baja pertenencia comunitaria de la población en la zona urbana. Chavis et al^7,8^ han aportado sustento sólido, mostrando la relación que existe entre el sentido de comunidad o pertenencia comunitaria y la participación comunitaria. Otros autores confirman lo reportado por Chavis et al,^18^ señalando que, en la medida que la pertenencia comunitaria contribuye a la participación, estos elementos en conjunto fortalecen la construcción de ciudadanía.^23^

La participación de la población fue favorable en el espacio rural, semejante a lo reportado en un estudio con adultos mayores en Chile.^15^

En esta investigación, se encontró escasa participación y compromiso de la autoridad local en la zona urbana, que es también reportado en otro estudio con enfoque ecosistémico en Ecuador.^3^ Coincidimos con estos autores, quienes señalan que la cultura política y el contexto son factores que influyen en la manera de proceder de las autoridades.

La participación de la población en el diagnóstico de salud se ubica a nivel local y tiene importancia debido a la identificación de los propios involucrados en sus problemáticas y soluciones. Menéndez menciona que las decisiones económicas y sociopolíticas que afectan la vida de las poblaciones son tomadas por organismos, autoridades e instancias políticas sin la participación de los grupos sociales mayoritarios.^19^ Entretanto, la participación en el nivel local es un proceso que puede contribuir a la construcción de ciudadanía y al fortalecimiento de actores sociales para que negocien frente al Estado,^5^ sin negar las dificultades y conflictos de poder, que pueden surgir durante el proceso entre la población y el Estado.

La población fue capaz de identificar, priorizar y comprometerse en la solución de problemas de salud en la zona rural. El diagnóstico de salud con enfoque de ecosalud establece una diferencia sustancial con respecto a los diagnósticos de salud tradicionales (administrativo, estratégico e ideológico), que parten de los servicios de salud y se enfocan en la programación de metas, objetivos y actividades dirigidas a la población por expertos, pero sin la participación de la población.^24,26^

Los resultados tienen sustento en la transdisciplina, participación, género/equidad, y concuerdan con los señalamientos de Dakubo,^9^ que sostiene que el abordaje actual de la salud pública se beneficia cuando se implementa una perspectiva holística que incorpora la complejidad del proceso salud-enfermedad.

Dakubo^10^ señala que la investigación basada en comunidad es un modelo que impulsa la participación de población, autoridades y equipo de investigación en las distintas fases del proyecto. En los diagnósticos de salud en este estudio se tomaron elementos de la investigación en comunidad, pero no se logró que la población urbana participara en todas las fases. Una herramienta utilizada para impulsar la participación de la población fue la cartografía social. Su uso obedeció para promover la participación y mejorar la comprensión del contexto local por los propios habitantes, y para encontrar soluciones a sus problemáticas.^2^

El enfoque de ecosalud aborda la equidad y el género como un solo principio. Primero, porque no se puede hablar de equidad sin abordar el género, y viceversa; segundo, porque dejar de lado estos temas es comprender parcialmente la situación de salud de las comunidades.^6,17^ Se integró la visión de hombres y mujeres en los diagnósticos de salud de la comunidad rural y parcialmente en la urbana.

Mertens^20^ et al consideran que el género es una categoría que muestra de manera diferencial la forma en que hombres y mujeres interactúan con el medio ambiente y la salud, y la manera como participan en los proyectos. Esto coincide con los resultados de la presente investigación.

Los retos identificados fueron: a) integrar en el instrumento la priorización de los problemas de los determinantes ambientales, que permitan reflexionar con la población las relaciones e interacciones sociales-ecológicas que afectan la salud, el bienestar y la sostenibilidad ambiental. b) diseñar estrategias y mecanismos que permitan impulsar la participación de la población en diversas zonas geográficas (rural/urbana) y en diferentes contextos socioculturales; c) sensibilizar a los tomadores de decisiones de los servicios de salud sobre la importancia de efectuar diagnósticos de salud poblacional con este enfoque; d) impulsar la integración no solo de las autoridades locales y municipales, sino también las estatales y nacionales en diagnósticos de salud con enfoque de ecosalud, con la finalidad de incidir en la formulación de políticas públicas.

El diagnóstico de salud poblacional es la herramienta principal del personal de salud y autoridades sanitarias para tomar decisiones, muchas veces limitadas por la forma en que se obtienen los datos (estadísticas de servicios de salud, sin tomar en cuenta a la población). Partimos de la premisa del diagnóstico de salud, con enfoque de ecosalud, como primer paso para procurar un acercamiento con las poblaciones y para generar conocimiento que conduzca a la acción de manera conjunta y que dé respuesta a sus necesidades.

Este trabajo es una primera aproximación de un diagnóstico de salud con base en el enfoque de ecosalud. Es necesario continuar realizando diagnósticos de esta naturaleza, ajustando las estrategias de acuerdo a las características de la población y zonas geográficas, a fin de profundizar en la concreción de los tres principios del enfoque que presentamos en este artículo.

## Footnotes

a Oportunidades es un programa federal mexicano enfocado a familias pobres que brinda apoyos en educación, salud y nutrición y la receptora de los recursos es la madre de familia.

b Instituto Nacional de Estadística y Geografía e Informática. II Conteo de población y vivienda 2010. México ( DF); 2010 [citado 2015 abr 19]. Disponible en: http://www3.inegi.org.mx/sistemas/mexicocifras/default.aspx?src=487&e=17

c Domínguez-Ortiz MG. Separación de los residuos sólidos urbanos, susceptibles a ser valorizados, que contribuya al mejoramiento del entorno de la colonia de Atlihuayan, municipio de Yautepec Morelos, bajo el enfoque ecosistémico [tesis]. Cuernavaca: Escuela de Salud Pública de México/Instituto Nacional de Salud Pública; 2012.

d Pacheco-Magaña LE. Manejo integral de los residuos sólidos para contribuir al control de enfermedades asociadas a estos, en la población de la localidad La Nopalera, Yautepec, Morelos bajo el enfoque de monitoreo participativo [tesis]. Cuernavaca: Escuela de Salud Pública de México/Instituto Nacional de Salud Pública; 2009.

e González C. Implementación de control biológico de moscas y propuesta para el manejo de excretas en la granja porcina de La Nopalera, Yautepec, Morelos [tesis]. Cuernavaca: Escuela de Salud Pública de México/Instituto Nacional de Salud Pública; 2010.

f Espinosa-Cárdenas F. Sexualidad y perspectiva de género: intervención educativa con jóvenes de La Nopalera [tesis]. Cuernavaca: Escuela de Salud Pública de México/Instituto Nacional de Salud Pública; 2009.

g Camarillo-Elizalde DG. Promoción de la salud y pertenencia comunitaria en escolares de Atlihuayan, Morelos [tesis]. Cuernavaca: Escuela de Salud Pública de México/Instituto Nacional de Salud Pública; 2012.

h Nieto Ayala M. Habilidades para la vida: prevención de adicciones en adolescentes de la telesecundaria Belisario Dominguez de Atlihuayan, Yautepec, Morelos [tesis]. Cuernavaca: Escuela de Salud Pública de México/Instituto Nacional de Salud Pública; 2012.
